# Variation in Ecophysiological Traits and Drought Tolerance of Beech (*Fagus sylvatica* L.) Seedlings from Different Populations

**DOI:** 10.3389/fpls.2016.00886

**Published:** 2016-06-22

**Authors:** Claudia Cocozza, Marina de Miguel, Eva Pšidová, L'ubica Ditmarová, Stefano Marino, Lucia Maiuro, Arturo Alvino, Tomasz Czajkowski, Andreas Bolte, Roberto Tognetti

**Affiliations:** ^1^Istituto per la Protezione Sostenibile delle Piante, Consiglio Nazionale delle RicercheSesto Fiorentino, Italy; ^2^BIOGECO, INRA, Univ. BordeauxCestas, France; ^3^Institute of Forest Ecology, Slovak Academy of ScienceZvolen, Slovak Republic; ^4^Dipartimento Agricoltura, Ambiente e Alimenti, Università degli Studi del MoliseCampobasso, Italy; ^5^Johann Heinrich von Thünen Institute, Institute of Forest EcosystemsEberswalde, Germany; ^6^Dipartimento di Bioscienze e Territorio, Università degli Studi del MolisePesche, Italy; ^7^The EFI Project Centre on Mountain Forests (MOUNTFOR), Edmund Mach FoundationSan Michele all'Adige, Italy

**Keywords:** water stress, ecophysiology, gas exchange, chlorophyll *a* fluorescence, European beech

## Abstract

Frequency and intensity of heat waves and drought events are expected to increase in Europe due to climate change. European beech (*Fagus sylvatica* L.) is one of the most important native tree species in Europe. Beech populations originating throughout its native range were selected for common-garden experiments with the aim to determine whether there are functional variations in drought stress responses among different populations. One-year old seedlings from four to seven beech populations were grown and drought-treated in a greenhouse, replicating the experiment at two contrasting sites, in Italy (Mediterranean mountains) and Germany (Central Europe). Experimental findings indicated that: (1) drought (water stress) mainly affected gas exchange describing a critical threshold of drought response between 30 and 26% SWA for photosynthetic rate and C_i_/C_a_, respectively; (2) the C_i_ to C_a_ ratio increased substantially with severe water stress suggesting a stable instantaneous water use efficiency and an efficient regulation capacity of water balance achieved by a tight stomatal control; (3) there was a different response to water stress among the considered beech populations, differently combining traits, although there was not a well-defined variability in drought tolerance. A combined analysis of functional and structural traits for detecting stress signals in beech seedlings is suggested to assess plant performance under limiting moisture conditions and, consequently, to estimate evolutionary potential of beech under a changing environmental scenario.

## Introduction

Warming-induced drought is threatening forest ecosystems worldwide, increasing water stress and mortality risk for trees (Allen et al., 2010). The vulnerability of plants to drought varies in dependence of stress severity, its duration, and the combination with other stresses (Niinemets, 2010). Intraspecific variation of tree response to drought, recently, has received increasing attention in the case of important forest species, such as *Fagus sylvatica* L. (beech; e.g., Borghetti et al., 1993; Tognetti et al., 1995; García-Plazaola and Becerril, 2000; Peuke et al., 2002; Aranda et al., 2015; Knutzen et al., 2015; Pšidová et al., 2015), in order to inform forest managers on adaptive capacities of populations for stress tolerance and decrease tree vulnerability to climate change.

Acclimation of trees to water deficit is the result of adaptive changes in plant development and ecophysiological processes, such as gas exchange, growth rate, and water relations (Sala et al., 2010). Drought-induced hydraulic limitation on carbohydrate use may prolong survival in plants under stress. However, if drought persists, reduced photosynthetic carbon assimilation due to stomatal closure (isohydric behavior) may promote carbon starvation, as carbohydrate demand continues for maintenance of osmoregulation, and plants fail to maintain hydraulic integrity (McDowell, 2011). If plants maintain their stomata open during drought (anisohydric behavior), hydraulic failure may occur, thus leading to mortality. Tree mortality may occur when drought has caused >50% loss of stem hydraulic conductivity, corresponding to −4.5 MPa in beech (Barigah et al., 2013). The capacity for adaptive changes to the environment may ultimately be critical in determining tree species survival under climate change (Aitken et al., 2008). Physiological responses, including adaptation and evolution to environmental changes, define phenotypic plasticity that can be assumed as the dominant underlying process with consequences on ecosystem functions (Hovenden and Vander Schoor, 2003; Thomas, 2011). A better understanding of geographic pattern and genetic variation in functional and structural traits of important tree species is essential for implementing adaptive forest management strategies to mitigate anticipated impacts of climate change on plant growth and drought tolerance.

Beech is a naturally dominant tree species in many European forests and sensitive to water deficit (Tognetti et al., 1995; Backes and Leuschner, 2000; Czajkowski et al., 2005; Bolte et al., 2007; Rose et al., 2009). The distribution of beech in Europe is characterized by high genetic diversity, resulting in high potential to adapt to changing environmental conditions (Dounavi et al., 2016). Acclimation to drought and heat stress in beech may occur after increasing levels of proline amino acid that plays as osmo-protectants to raise the osmotic pressure and thus maintain membrane integrity and stabilize proteins (Rennenberg et al., 2006). Beech can also respond to water stress through decrease in photosynthetic efficiency and light sensitivity of the photosynthetic apparatus (Tognetti et al., 1995, 1997; Peuke et al., 2002). In southern Europe, the recent decline in basal area increment of beech has been linked to decreasing water availability (Jump et al., 2006; Piovesan et al., 2008), which can affect carbon dynamics and sequestration potentials at the southern limit of this species distribution (Tognetti et al., 2014). However, this is not a general response and positive growth (tree-ring width) in beech at Mediterranean latitudes has been observed (Tegel et al., 2013). In central Europe, the extreme 2003 drought has not been found to induce dramatic growth reduction in beech (Leuzinger et al., 2005; Van der Werf et al., 2007), while recording large reductions in gross primary production (GPP) in temperate forests (Ciais et al., 2005; Granier et al., 2007). Nevertheless, the radial growth of beech has been reported to be reduced in the context of more frequent and intensive drought extremes (Beck and Heußner, 2012). Although beech is considered vulnerable to climate change (Ohlemuller et al., 2006; Geßler et al., 2007; Scherrer et al., 2011), its distribution has been modulated, by forest conversion from coniferous forests to mixed stands (Fischer et al., 2013). Beech populations have been able to adapt to environmental changes depending on the level and distribution of genetic variation within and between these populations and their phenotypic plasticity (Bresson et al., 2011; Vitasse et al., 2014).

Plasticity of functional traits plays a determinant role in plant response to different environments, providing relevant information in resistance and resilience of beech to global warming (Stojnić et al., 2015). Köcher et al. (2009) found that beech adjusted the vitality and productivity as function of the drought sensitivity of stem growth and leaf and root production, and the success of rejuvenation under a drier climate. Furthermore, changes in photosynthetic characteristics induced by water stress were similar between beech seedlings originated from different sites, but the intensity of change was dependent on the origin (Pšidová et al., 2015). However, phenotypic differences in populations are related to the different directional selection defined by environmental conditions and evolutionary processes (Poorter et al., 2010). Disturbances may also lead to higher genotypic variability at the population level (Borghetti et al., 1993). Beech may exhibit intraspecific variation in drought resistance strategies characterized by varying degree of anisohydricity or isohydricity. A prompt stomatal reaction in beech populations from drier sites may be expected, vs. low stomatal regulation in the plants from mesic conditions (Aranda et al., 2015). Differences in the sensitivity of photosystem II (PSII) to drought among populations may also occur, because those with a drought-avoiding strategy limit carbon assimilation more than drought-tolerant seed sources.

Within this framework, provenance trials represent a valuable tool for assessing adaptive potential to a changing environment (Stojnić et al., 2015). Common garden investigations are carried out to test hypotheses on the geographic variation of adaptive traits in tree species, and study the relationships between trait variability and seed source environments (i.e., Sork et al., 2013). In the present study, beech from a wide range of European populations was selected throughout its native range, considering the role of local adaptation and phenotypic plasticity of this species (according to Bolte et al., 2016). We hypothesized that divergence in the plasticity of the response to environmental conditions should have occurred in beech populations originating from different localities. Specific hypothesis were: (1) population differentiation of traits will be moderate to high based on beech distribution; (2) population traits will match seed source climate and will reflect local adaptation (higher water use efficiency in populations from drier sites); (3) populations will distribute in European sites based on morphological and physiological characteristics. Two common garden experiments were carried out in Germany and Italy to quantify geographic variation of adaptive traits in beech populations and investigate relationships between trait variability and seed source, determining the effects of water stress on assimilation rate, predawn water potential, chlorophyll *a* fluorescence and chlorophyll content, (**Experiment 1**) and the assimilation potential (e.g., gas exchanges and leaf traits) (**Experiment 2**).

## Materials and methods

### Plant material

Beech populations were collected through the pan-European EU Cost STReESS network, from seven sites in six European countries. Seeds were collected in the fall of 2013 from numerous maternal families in forest stands throughout the native range of the species, representing a gradient in mean air temperature and rainfall (Table 1). Uniformly sized seeds of each population were surface-sterilized by soaking in 3% sodium hypochlorite for 5 min and rinsing with deionized water. Therefore, a stratification procedure was performed: (1) the seed moisture content was reduced to about 8% of the fresh seeds' moisture content (e.g., by storing them about 1 week at a dry and cool place); (2) seeds were preserved in plastic bags in a freezer at −5°C until the mid of February 2014 (stratification by frost); (3) the seed moisture was increased (using a water sprayer) at a temperature of 3–5°C; (4) as soon as the first little sprout was visible, the seedlings were transplanted in pots. Seedlings obtained from seeds were planted in pots.

**Table 1:** **Location, average of mean annual air temperature (°C), and total annual rainfall (mm) at the seven sites where the seedlings originated**.

**Altitude order**	**PV1**	**PV2**	**PV3**	**PV4**	**PV5**	**PV6**	**PV7**
Country	Denmark	Germany	France	France	Romania	Bosnia	Spain
Provenance	*Stenderup Midtskov*	*Sellhorn*	*Crecy*	*Montagne Noir*	*Valea Baronului*	*Nevesinje*	*Erro*
Longitude	9.65	9.93	1.88	2.22	21.68	18.13	−1.47
Latitude	55.47	53.35	50.225	43.50	44.77	43.27	43.00
Altitude (GIS Interpol.)	18	86	30	341	445	862	931
Mean annual air temperature (°C)	7.7	8.2	10.5	12.4	9.3	9.6	9.1
Total annual rainfall (mm)	720	748	637	791	722	1199	1166

A different level of success of seed germination was observed, thus different populations were considered in each experiment. Seven populations (“Denmark” PV1, “Germany” PV2, “France-Crecy” PV3, “France-Montagne Noir” PV4, “Romania” PV5, “Bosnia” PV 6, and “Spain” PV 7) were tested in Experiment 1 in the North-East of Germany. Four populations (PV1, PV3, PV4, and PV5) were used in Experiment 2 in Central Italy.

### Experimental set-up

Two common garden studies were carried out accurately to estimate differentiation under diverse environmental conditions determined by different latitude and elevation between sites (Central Europe, Germany; Mediterranean mountains, Italy). The two experiments took place in greenhouses; plants were subjected to the ambient light without additional illumination; plants were grown in cylindrical PVC pots (1.4 L) filled with a mixture of 70% silty-sand soil (grain size 0–2 mm) and 30% peat-based substrate amended with 2 kg m^−3^ Osmocote (NPK 14:13:13+7SO_3_, plus micro elements).

Twenty replicate seedlings for each population per treatment were designated, by selecting homogenous “matched pairs” based on visual comparison. Well-watered (control) seedlings were regularly watered with tap water to field capacity throughout the experiment. Seedlings subjected to drought treatment were submitted to progressive drought by withdrawing irrigation, when full leaf unfolding had occurred. Initial soil water content was estimated by weighing the samples before and after oven drying at 105°C for 48 h. Pots were initially watered to saturation. After excess water has drained away field capacity (FC, Blume et al., 2016) was reached at −0.06 MPa soil water potential (pF 1.8). Subsequent changes in pot weight were attributed to changes in soil water content.

A group of 20 seedlings (control) was maintained near to FC by daily watering; whereas, water supply was suspended for those seedlings (20 individuals) subjected to drought treatments. Since watering interruption, each pot was weighed three times per week, in order to estimate the plant available water content (θ_AWC_), using the following Equation (1, cf. Veihmeyer and Hendrickson, 1927):

(1) $$\begin{array}{l} \theta_{\text{AWC}}=\theta_{\text{FC}}-\theta_{\text{PWP}} \end{array}$$

where θ is the soil water content (g) at FC (pF 1.8 ≈ −0.06 MPa soil water potential) and at the visual loss of leaf turgor (commonly defined permanent wilting point in agricultural plants; PWP, pF 4.2 ≈ −1.5 MPa soil water potential). The permanent wilting point (PWP = −1.5 MPa) is used as a conventional threshold for determining the soil water availability (SWA) within the entire effective rooting depth according to the concept of Reid et al. (1984). The PWP was derived for agricultural plants, and young trees like European beech may deplete water resources near to the fine roots at lower soil water potentials than the PWP.

The residual soil water availability (SWA; %) is defined as the proportion of available soil water content (θ_AWC_) during drought treatment referred to the initial value under condition of field capacity.

During the drought experiment the pots were placed at random position and re-randomized after every watering event. Each treatment group was ideally represented by 20 individuals corresponding to replicate seedlings for each population per drought treatment. The pots were allowed to reach different thresholds of drought (50, 40, and 30% SWA) at different time.

Plant responses to extreme drought were studied focusing on gas exchange and water relations, and anatomical traits. We put specific emphasis on the assimilation ability and leaf anatomical traits determining gas exchange and transpiration processes.

Leaf gas exchange was measured on one leaf of five seedlings per treatment once per week, using a portable gas exchange system (GFS-3000, Heinz Walz GmbH Germany) in Germany, and using a LI-COR photosynthesis system (LI-6400; LI-COR Inc., Lincoln, NE, USA) in Italy. The adjusted values were: leaf/cuvettes temperature 25°C, (Air-to-leaf), Vapor Pressure Deficit 13.8 −16.1 (Pa/kPa), CO_2_ concentrations 360 ppm, flow 750 μmol s^−1^, and the light intensity (LED Light Source 3040-L) 1500 μmol m^−2^ s^−1^. Measurements (assimilation rates and water vapor conductance) were taken between 09:30 and 14:30 (UT) on fully expanded un-shaded leaves and calculated according to von Caemmerer and Farquhar (1981). Instantaneous photosynthetic rate and corresponding stomatal conductance, ratio of intercellular (C_i_) to ambient (C_a_) CO_2_ concentration (C_i_/C_a_), and ratio of photosynthetic rate (A) to stomatal conductance (A/g_s_), namely leaf intrinsic water use efficiency (WUE), were measured at light intensity of 1000 μmol m^−2^ s^−1^ (LED source, red blue 6400-02B) and with a controlled flow of 400 μmol s^−1^ of ambient air with CO_2_ concentration fixed at 400 μmol mol^−1^. Air temperature and relative humidity were maintained close to ambient values.

For specific insights in plant water status variation during treatment predawn (1 h before the start of the daily light regime) leaf water potential (Ψ_pd_; MPa) was measured in three seedlings per treatment per provenance to obtain the mean soil water potential next to the roots, closely correlated to the relative transpiration rate (Améglio et al., 1999). Predawn leaf water potential was measured between 1:00 and 5:00 (UT). Exact measurement times varied over the course of the experiment, as the predawn time changed during the experiment. One leaf per five seedlings were detached and rapidly enclosed in a Scholander-type pressure chamber (SKPM1400, Skye Instruments, Llandrindod Wells, UK).

### Experiment 1

The Experiment 1 was carried out in late summer 2014 by the Thünen Institute of Forest Ecosystems—University of Sustainable Development (HNE) in Eberswalde (52°49′28″ N 13°47′29″ E, 30 m a.s.l.). Within the treatment period relative air humidity averaged 69%, with a minimum of 30% and a maximum of 88%. Air temperature ranged between 11°C (minimum during night) and 31°C (maximum during day), and attained a mean of 19.0°C.

Seedlings reached the ontogenic stage of four fully expanded leaves in 06 August 2014, when the water irrigation was interrupted in mid-summer (06 August 2014) until desiccation of seedlings (06 October 2014), which defined the end of experiment (the duration of experiment was 61 days). Root collar diameter, plant height, leaf area and leaf number were measured at the beginning of the experiment in control seedlings.

Fast chlorophyll *a* fluorescence kinetics were determined using the chlorophyll fluorimeter Handy PEA (Hansatech, Instruments Ltd, UK). The following parameters were measured: minimal (F_0_), maximal (F_m_), and variable (F_v_ = F_m_ − F_0_) fluorescence of dark-adapted leaves; the dark-adapted leaf is illuminated with weak modulated measuring light to give the background fluorescence level in the dark (F_0_); application of a saturation pulse allows measurement of the maximum fluorescence level in the dark (F_m_). The maximal efficiency of PSII photochemistry was calculated as F_v_/F_m_. The performance index, PI, indicator of sample vitality reflecting the energy cascades, was also determined. Relevant fluorescence parameters were measured using the portable chlorophyll fluorometer MINI-PAM (Walz, Heinz Walz GmbH, Effeltrich, Germany). Rapid light curves (RLCs) were recorded after each chlorophyll fluorescence kinetics measurement: yield (Φ_PSII_), the photochemical yield of photosystem II; ETR, the relative rate of electron transport; qP, the coefficient of photochemical quenching; qN, the coefficient of non-photochemical quenching; NPQ, the non-photochemical quenching. Relative chlorophyll content of the leaves was estimated by portable chlorophyll meter (CL-01, Hansatech, Instruments Ltd., Kings Lynn, UK). The results were expressed as chlorophyll index (Chl index; Cassol et al., 2008). Values of Chl index were estimated by device on the basis of the absorbance at 620 and 940 nm.

### Experiment 2

The Experiment 2 took place during mid-summer 2014 at the University of Molise in Pesche (41°37′00″ N 14°17′00″ E, 732 m a.s.l.). During the experiment, relative humidity ranged between 60 and 70% and air temperature between 15°C (night) and 30°C (day), with a mean of 22.5°C. The light intensity at the plant level never exceeded 1000 μmol photons m^−2^ s^−1^ in sunny days (>80% of the days during which the experiment was carried out), whereas the vapor pressure deficit (VPD) rarely exceeded 2 kPa. In both experiments, the plants were subjected to the ambient light without additional illumination.

Due to the different climatic conditions, the seasonal drought is stronger, occurs earlier and lasts longer in Italy than in Germany. Therefore, seedlings reached the ontogenic stage of four fully expanded leaves in 26 June 2014 in Italy, when water irrigation was interrupted in early summer until desiccation of seedlings in 26 July 2014 (the duration of experiment was 30 days).

Light response curves (A/Q) and intercellular CO_2_ response curves (A/C_i_) were measured on full expanded leaves from five seedlings grown in control conditions at the end of experiment using a portable photosynthesis system (LI-6400, Li-Cor, Lincoln, NE; according to Tognetti et al., 2004). Light response curves were obtained by measurements at Q-values of 2000, 1500, 1000, 500, 200, 100, 50, 20, and 0 μmol m^−2^ s^−1^ (LED source, red blue 6400-02B). Measurements were recorded automatically at each set point when A had equilibrated; irradiance was changed at intervals of 120–200 s. The CO_2_ entering the cuvette was adjusted to maintain a chamber CO_2_ concentration ([CO_2_]) of 400 μmol mol^−1^. The response of leaf A to Q was modeled by a non-rectangular hyperbola where the initial slope was apparent quantum efficiency (φ), light compensation point (Γ_l_), and apparent dark respiration (R_d_) were estimated from axis intercepts, and the light-saturated maximum photosynthetic rate (A_max_) was the upper asymptote. All parameters were determined by fitting data to the model function (Prioul and Chartier, 1977). See Tognetti et al. (2004) for further details on curve fitting and equations.

The CO_2_ response curves were obtained by changing the [CO_2_] entering the cuvette from 50 to 800 μmol mol^−1^ with an external CO_2_ cartridge mounted on the LI-6400 console and automatically controlled by a CO_2_ injector. The CO_2_ assimilation rate was first measured by setting the reference [CO_2_] near ambient (400 μmol mol^−1^) and then at 300, 200, 100, 50, 400, 400, 600, and 800 μmol mol^−1^. Gas exchange was determined at each step after exposure of the leaf to the new [CO_2_], waiting for A to reach equilibrium, which was typically < 3 min; Q was maintained at 1000 μmol m^−2^ s^−1^. The response of leaf A to Ci was analyzed according to the mechanistic model of CO_2_ assimilation proposed by Farquhar et al. (1980) and subsequently modified by Sharkey (1985). A non-linear regression technique was used to estimate R_day_, the maximum rate of carboxylation (V_cmax_), the light-saturated rate of electron transport (J_max_), and the rate of triose phosphate utilization for sucrose and starch synthesis (TPU; Sharkey, 1985; Wullschleger, 1993). Description of parameter estimates can be found in Tognetti et al. (2004). The entire leaf area entered the cuvette, limiting errors associated with area determination and the occurrence of a patchy distribution of stomata.

Leaf thickness was measured (lower and upper epidermis, palisade, and spongy parenchyma) on transversal sections (four measurements in each of five plants were averaged). Stomatal density (number of stomata per mm^2^ of leaf area), and the average of polar (length) and equatorial (width) stomatal size, and distance between guard cells were determined. Three regions were measured in the median leaf blade, summarizing 30 observations per population. Structural leaf traits were measured on three leaves of three plants per population on all plants at the beginning of the experiment using the uppermost fully expanded leaves. Leaf portions (1–2 cm in diameter) were fixed with 3% glutaraldehyde (v/v) in 0.1-M phosphate buffer (pH 7.2) for 6–8 h under 4°C, post-fixed in 1% osmium tetroxide for 1 h, and immersed in 0.1-M phosphate buffer (pH 7.2) for 1–2 h. The samples were then dehydrated in a graded ethanol series (50, 60, 70, 80, 90, 95, and 100%) with a last wash in acetone for a better CO_2_ substitution during the dehydration procedure at a pressure of 1200 bars. Dry tissue samples were coated with gold in a sputter coater and observed in a scanning electron microscope (SEM Zeiss DSM 940A, Oberkochen, Germany) operated at 10 keV (according to Hultine and Marshall, 2001).

### Data analysis

Because all seedlings in each experiment shared the same substrate conditions (nutrient availability, soil texture, etc.), we assumed a significant main effect of imposed dry-down conditions on phenotypic plasticity of physiological traits. Data were examined for assumptions of the homogeneity of variance and normality and were found to conform to model requirements. Variations in leaf gas exchange parameters during the experiment were evaluated by two-way repeated measures ANOVA with “population,” as random factor, and “soil water availability,” as fixed factor. One-way ANOVA was performed to analyze the effect of population on A/Ci and A/Q curve parameters, root collar diameter, plant height, leaf area and number, and microscopic leaf traits. Statistical analysis was conducted with OriginPro version 8.5.1 (OriginLab, Northampton, MA). Statistical analyses were separately performed considering the different experimental period between Germany and Italy.

## Results

### Experiment 1

Beech seedlings differed in the root collar diameter, plant height, and leaf number (*p* < 0.0001) between populations in control conditions (Table 2). Root collar diameter was higher in German (PV2), French (PV3 and PV4), and Bosnian (PV6) populations; plant height in French (PV3 and PV4), Romanian (PV5), and Bosnian (PV6) populations; leaf number in German (PV2), French (PV3), Romanian (PV5), and Bosnian (PV6) populations (Table 2).

**Table 2:** **Root collar diameter, plant height, leaf area, and number for beech seedlings at the beginning of the water-stress experiment**.

	**PV1**	**PV2**	**PV3**	**PV4**	**PV5**	**PV6**	**PV7**	**ANOVA**	***F*-value**	***P*-level**
Root collar diameter (mm)	2.00 (0.00)	2.44 (0.09)	2.25 (0.10)	2.45 (0.11)	2.00 (0.00)	2.75 (0.10)	2.00 (0.00)	Population	4.696	0.000
Plant height (cm)	14.67 (0.32)	12.44 (0.62)	17.50 (0.40)	14.92 (0.76)	15.42 (0.47)	16.77 (0.79)	13.80 (0.45)		7.568	0.000
Leaf area (cm^2^)	77.11 (5.47)	99.62 (12.70)	99.44 (12.19)	69.99 (11.50)	111.25 (16.12)	110.43 (7.47)	90.12 (11.61)		1.918	0.098
Leaf number (*n*)	9.50 (0.90)	13.13 (2.38)	12.75 (1.50)	4.40 (0.57)	15.00 (1.64)	15.63 (0.86)	9.63 (2.27)		5.171	0.000

*The values for the mean (± standard error), and the statistical analysis of the populations (ANOVA test) (F-value, P-level) are shown (experiment 1)*.

**Figure 1:**
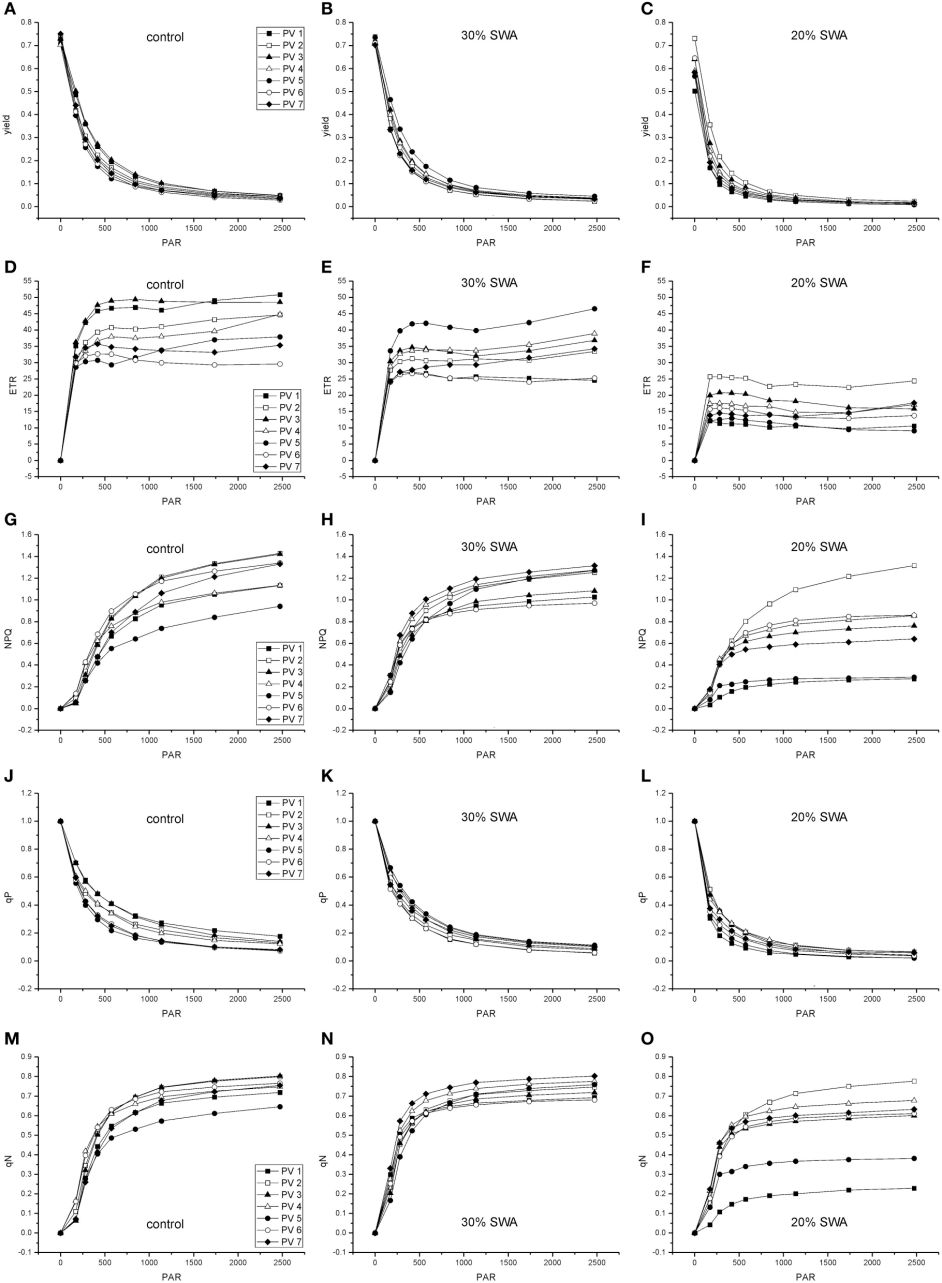
**Effects of drought treatment on rapid light response curves of the maximal yield of photochemical energy conversion (yield; A-C), the relative photosynthetic electron transport rate (ETR; D-F), the quantum yield of regulated energy dissipation of PSII (NPQ; G-I), the coefficient for photochemical quenching (qP; J-L), the coefficient for non-photochemical quenching (qN; M-O) in beech seedlings in days of drought treatment (experiment 1)**.

**Figure 2:**
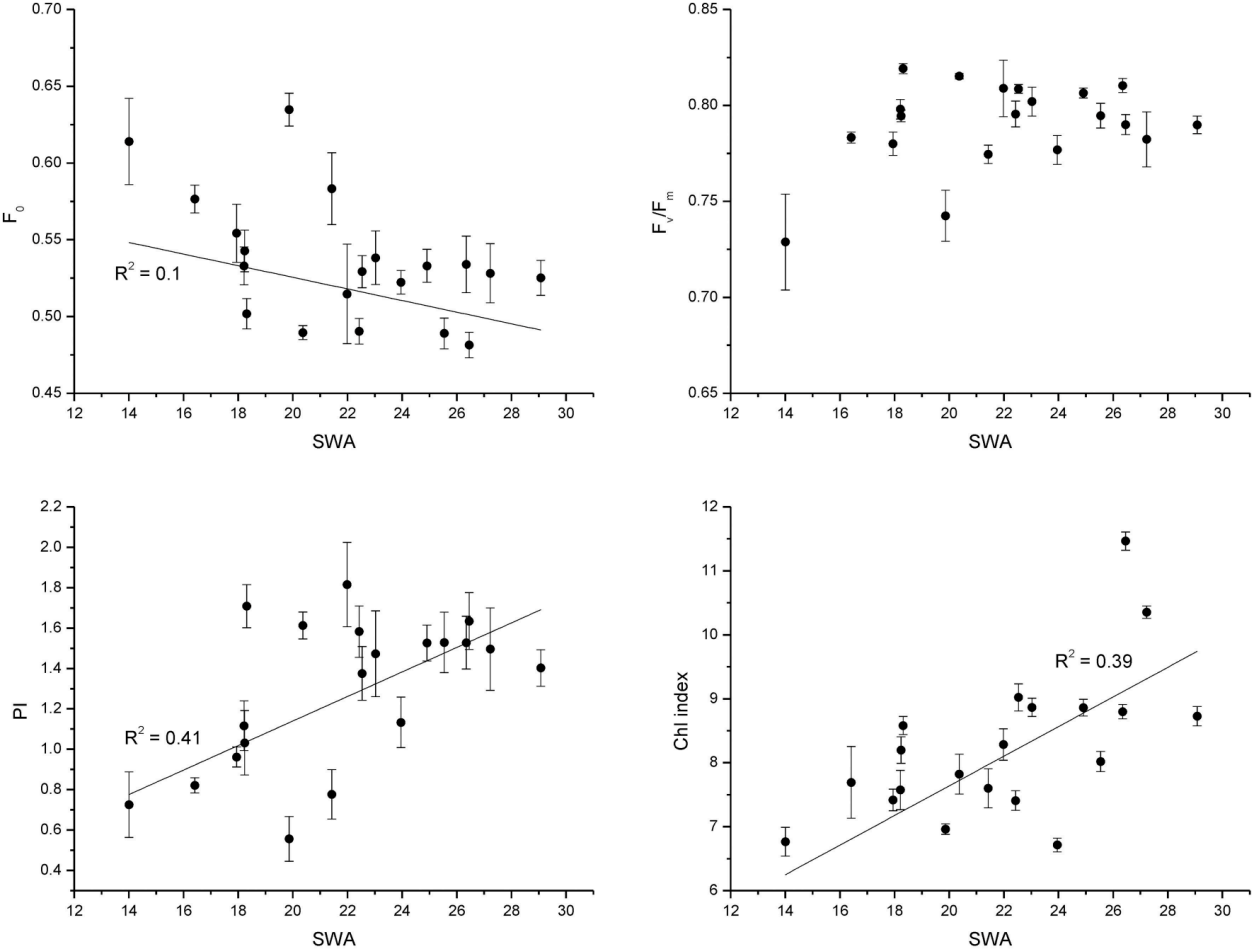
**Fluorescence traits (F_**0**_), the minimal Chl fluorescence; (F_**v**_/F_**m**_), the maximal efficiency of PSII photochemistry; (PI), the performance index; (Chl index), chlorophyll index, following the soil water availability during the experiment (experiment 1)**.

The parameters derived from rapid light curves decreased with decreasing SWA (Figure 1). Yield and qP showed the same decreasing pattern in all populations. After 48 days of drought treatment, when 20% of SWA was reached, ETR, NPQ, and qN were reduced in comparison with control; however, PV2 showed higher values and PV1 and PV5 than other populations (Figure 1). The reduction of SWA showed an increasing trend for F_0_, and decreasing pattern for PI and Chl index; whereas, F_v_/F_m_ was not substantial affected by water stress (Figure 2).

**Figure 3:**
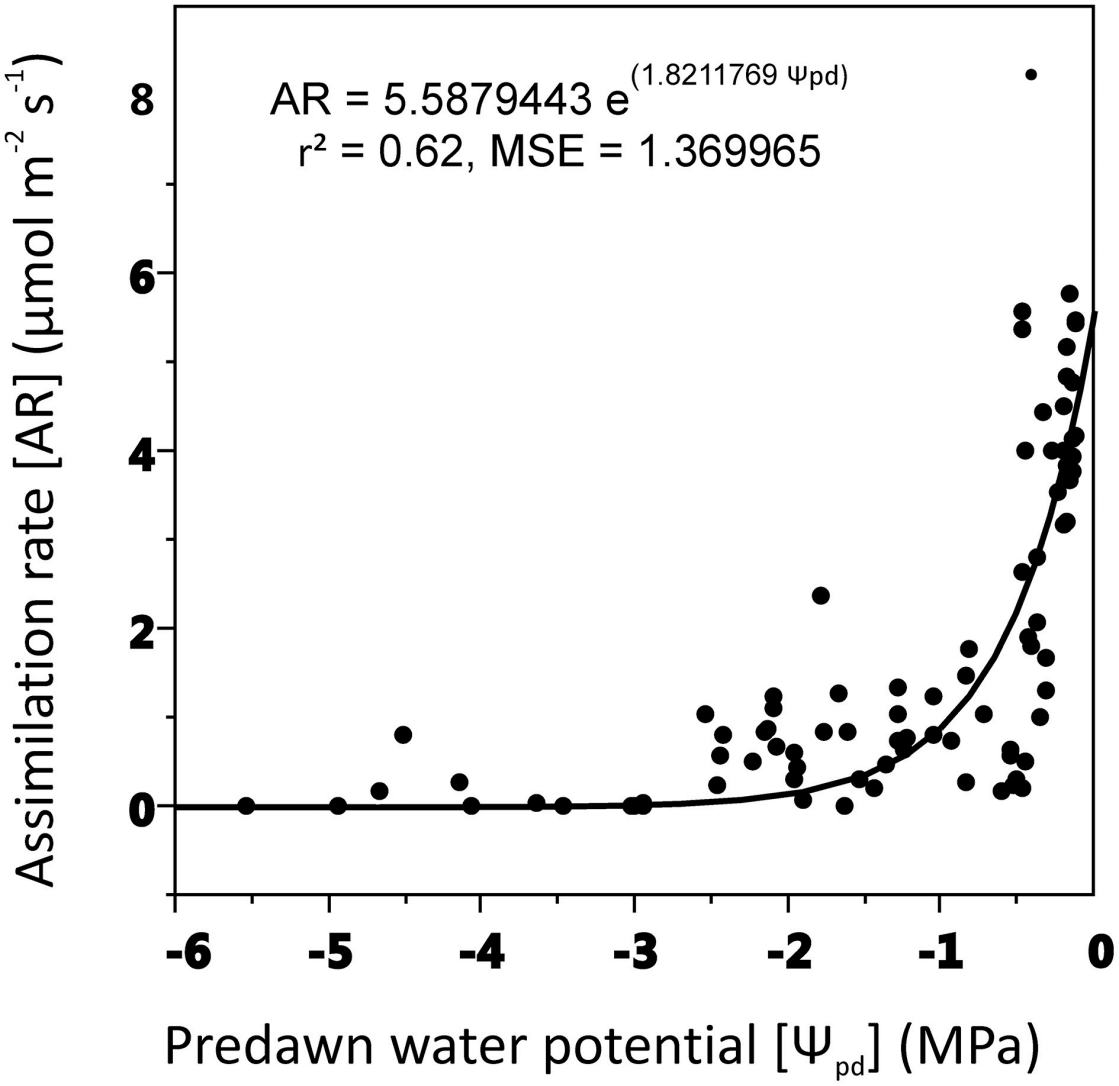
**Relationships between assimilation rate (AR) and shoot predawn water potential (Ψ_**pd**_)**. Non-linear regression model (exponential function) was used for curve-fitting analysis (experiment 1).

Assimilation rate decreased as predawn leaf potential became more negative with a steep drop at leaf water potential close to −2 MPa (Figure 3).

### Experiment 2

Leaf traits were significantly different among populations in control conditions (Table 3). However, leaf traits did not show a common pattern among populations, suggesting complex phenotypic effects driven by prevailing environmental conditions at the geographical origin (Table 3). The lowest values of stomatal density and spongy mesophyll thickness were found in PV4 and PV5, of mesophyll thickness in PV4, of stomatal length (polar size) in PV3, and of stomatal width (equatorial size) in PV1.

**Table 3:** **Structure of fully expanded foliage (harvested at the beginning of the experiment) of seedlings for each population grown in control conditions**.

	**PV1**	**PV3**	**PV4**	**PV5**	***F*-value**	***p*-level**
Stomatal density (n mm^−2^)	7.00 (0.29)	6.60 (0.20)	5.80 (0.21)	4.60 (0.26)	20.63	0.000
Mesophyll (μm)	68.84 (2.27)	72.20 (0.92)	58.15 (1.53)	64.96 (1.15)	16.14	0.000
Palisade (μm)	33.84 (0.85)	33.15 (0.82)	26.68 (0.61)	34.04 (0.49)	26.54	0.000
Spongy (μm)	23.19 (2.02)	27.52 (0.87)	21.21 (1.20)	19.99 (1.42)	5.659	0.002
Polar size (μm)	8.97 (0.50)	8.23 (0.24)	8.44 (0.49)	10.29 (0.39)	5.316	0.003
Equatorial size (μm)	3.51 (0.20)	3.71 (0.20)	3.97 (0.18)	4.46 (0.36)	2.982	0.039
Distance between guard cells (μm)	11.37 (0.20)	12.15 (0.33)	11.51 (0.20)	11.58 (0.20)	2.23	0.095

*Values are means ± standard error for population (n = 5 seedlings). Significance (F-value; P-level) of population effects is shown (experiment 2)*.

Photosynthetic response curves to internal CO_2_ and irradiance (analyzed in control plants) did not show differences among populations (Table 4).

**Table 4:** **Populations effects on photosynthesis (A) response curves to internal [CO_**2**_] (C_**i**_) (A/C_**i**_) and to irradiance (Q) (A/Q)**.

	**PV1**	**PV3**	**PV4**	**PV5**	**ANOVA one way**	
					***F*-value**	***P*-level**
**A/Ci CURVE PARAMETERS**						
R_day_	2.67 (1.71)	1.46 (0.07)	−0.03 (0.08)	0.83 (0.41)	2.00	0.232
V_cmax_	26.65 (14.32)	21.61 (1.89)	20.58 (4.37)	27.25 (0.33)	0.17	0.912
J_max_	31.16 (14.74)	21.10 (2.93)	20.39 (3.45)	26.42 (0.66)	0.39	0.762
TPU	2.86 (0.58)	2.51 (0.16)	2.67 (0.36)	2.77 (0.20)	0.17	0.911
**A/Q CURVE PARAMETERS**						
A_max_	5.49 (0.43)	5.21 (0.91)	6.07 (1.37)	5.27 (0.83)	0.26	0.854
φ	0.09 (0.01)	0.09 (0.00)	0.09 (0.02)	0.10 (0.01)	0.63	0.614
R_d_	−0.19 (0.06)	−0.31 (0.09)	−0.25 (0.20)	−0.21 (0.13)	0.05	0.983
Γ_l_	2.67 (1.63)	2.67 (1.63)	1.31 (1.60)	2.65 (1.62)	0.26	0.852

*Measurements were made on fully expanded leaves of control plants. Values are the means ± standard error for population (n = 5 seedlings). Significance (F-value; p-level) of population effects for A/Ci and A/Q curve parameters are presented. R_day_, daytime respiration (μmol CO_2_ m^−2^ s^−1^); V_cmax_, maximum rate of carboxylation (μmol CO_2_ m^−2^ s^−1^); J_max_, light-saturated rate of electron transport (μmol electrons m^−2^ s^−1^); TPU, rate of triose phosphate utilization (μmol CO_2_ m^−2^ s^−1^); A_max_, maximum photosynthetic rate (μmol CO_2_ m^−2^ s^−1^); φ, apparent quantum efficiency (μmol CO_2_ μmol^−1^ photons); R_d_, apparent dark respiration (μmol CO_2_ m^−2^ s^−1^); and Γ_l_, light compensation point (μmol photons m^−2^ s^−1^) (experiment 2)*.

Photosynthetic rate, C_i_/C_a_ and the ratio photosynthesis and stomatal conductance (A/g_s_) significantly differed among populations (*P* < 0.001) (Table 5). Decreasing SWA impacted all considered ecophysiological traits (*P* < 0.001), as well as the interaction population x treatment (*P* < 0.001).

**Table 5:** **Daytime gas exchange under saturating light conditions of seedlings for each population grown in different drought thresholds (treatment)**.

**Gas exchange parameter**	**SWA (%)**	**PV1**	**PV3**	**PV4**	**PV5**	**ANOVA**	***F*-value**	***P*-level**
**PHOTOSYNTHESIS RATE (A) (μmol m^−2^ s^−1^)**								
	30	1.10 (0.55)	0.36 (0.24)	1.36 (0.65)	0.52 (0.50)	Population	8.087	0.000
	40	4.04 (0.45)	3.35 (0.59)	2.51 (1.06)	3.56 (0.52)	Treatment	59.940	0.000
	50	5.51 (0.29)	4.63 (0.44)	3.81 (0.60)	4.19 (0.42)	Population × Treatment	34.806	0.000
	Control	5.26 (0.12)	4.03 (0.20)	4.35 (0.32)	4.30 (0.30)			
**STOMATAL CONDUCTANCE (gs) (mol m^−2^ s^−1^)**								
	30	0.01 (0.00)	0.01 (0.00)	0.02 (0.00)	0.01 (0.00)	Population	1.114	0.344
	40	0.06 (0.01)	0.05 (0.01)	0.04 (0.02)	0.05 (0.01)	Treatment	34.965	0.000
	50	0.07 (0.00)	0.07 (0.01)	0.06 (0.01)	0.06 (0.01)	Population × Treatment	18.313	0.000
	Control	0.06 (0.00)	0.05 (0.00)	0.06 (0.01)	0.05 (0.00)			
**C_i_/C_a_ (μmol mol^−1^)**								
	30	0.69 (0.08)	0.89 (0.06)	0.71 (0.08)	0.87 (0.07)	Population	6.372	0.000
	40	0.61 (0.03)	0.70 (0.03)	0.71 (0.04)	0.65 (0.04)	Treatment	21.689	0.000
	50	0.61 (0.02)	0.66 (0.03)	0.70 (0.02)	0.63 (0.03)	Population × Treatment	14.201	0.000
	Control	0.57 (0.02)	0.63 (0.02)	0.64 (0.02)	0.59 (0.02)			
**A/gs (μmol mol^−1^)**								
	30	66.88 (21.33)	15.27 (15.40)	61.60 (19.55)	19.55 (17.95)	Population	6.148	0.000
	40	85.23 (7.52)	50.90 (16.97)	60.35 (10.84)	73.66 (10.88)	Treatment	21.008	0.000
	50	82.79 (3.95)	73.78 (8.23)	63.97 (5.59)	78.00 (7.14)	Population × Treatment	13.670	0.000
	Control	95.61 (4.06)	82.75 (5.29)	78.22 (4.33)	92.97 (4.93)			

*Photosynthetic rate (A), stomatal conductance (g_s_), ratio of intercellular (C_i_) to ambient (C_a_) [CO_2_] (C_i_/C_a_), and leaf intrinsic WUE (A/g_s_) were measured on the first fully expanded leaf (LPI 5) (measurements made at 1000–1200 h). Values are means (± standard error) for population (n = 5 seedlings). Significance of treatment effect (drought treatment expressed at SWA level; ANOVA) for each variable and their interaction is indicated by F-value and P-level (experiment 2)*.

**Figure 4:**
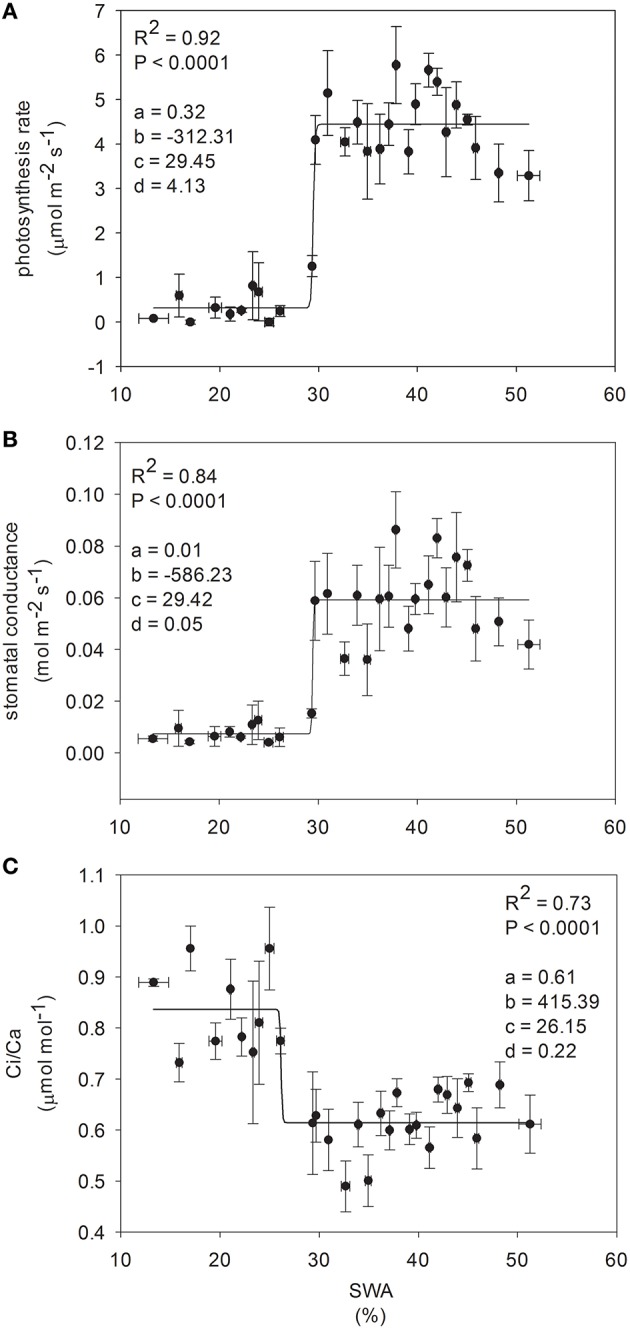
**Relationship between ecophysiological traits and soil water availability (each point corresponds mean of five plants)**. Non-linear regression model was used for curve-fitting analysis, such as a four parameter logistic equation, where: **(A)** is the minimum asymptote; **(B)** is the hill slope; **(C)** is the inflection point; **(D)** is the maximum asymptote; global goodness of fit is defined by R-squared, and significance of the regression by p-level (experiment 2).

Gas exchange was related to SWA through a sigmoidal pattern, describing a threshold-type transition from one phase to the next (Figure 4). The inflection point of the function was observed between 30 and 26% SWA (29.45, 29.42, and 26.15% SWA, for photosynthetic rate, stomatal conductance and Ci/Ca, respectively).

## Discussion

### Differences among populations

Beech seedlings from seeds collected across a climatic gradient were monitored in two common garden experiments conducted in Germany and Italy to quantify the geographic structure of variation in ecophysiological traits and the adaptation potential of populations to drought stress. We hypothesized that differentiation of tolerance/avoidance traits in beech progenies was constitutively linked to the seed source, and tested whether population-specific functional and structural responses to drought stress were revealed when seedlings were exposed to decreasing soil water availability.

The study highlighted intraspecific differentiation in plant performance when seedlings were not exposed to constraints (Tables 2–4), which was related to phenotypic variation among populations (e.g., Thiel et al., 2014). Beech has a wide area of distribution and the gradual change in ecophysiological traits and environmental gradients exhibited by different populations can shape local adaptation. Netzer et al. (2016) found that Greek populations were more tolerant to drought stress than German ones, while a Bulgarian population was the most sensitive. These results confirm once again the relevance of seed source for assessing the response of beech to drought stress. The French population (PV3) showed a distinct behavior in morphological traits; whereas, Danish (PV1) and Spanish (PV7) populations showed the lowest plant height, collar diameter, and leaf number (Table 2). Well-watered conditions resulted in higher plant height in French, Romanian, and Bosnian populations, and higher leaf number in German, French, Romanian, and Bosnian populations, suggesting higher plant growth potential in French, Romanian, and Bosnian populations, but failing to identify a clear population-related trait. Contradictory results on growth differentiation between beech populations with different native precipitation regimes are reported in literature (e.g., Tognetti et al., 1995; Knutzen et al., 2015). Indeed, genetic differentiation between beech stands was found to be located mainly within stands and not between stands within the distribution range of this species (Buiteveld et al., 2007; Meier and Leuschner, 2008). Even though, clear genetic differentiation also among stands based on adaptive genetic markers for populations coming from different climatic conditions was found by Sander et al. (2000) and Dounavi et al. (2016). Results of photosynthetic response curve to internal CO_2_ concentration and irradiance did not show differences among populations, highlighting common assimilation potential and photosynthetic capacity across populations (Table 4). These results pointed to the lack of intraspecific differences in these traits among populations related to drier or moister native conditions, as observed by Knutzen et al. (2015). The impact of the last glacial period on the areal distribution of beech and the preferred selection of seed sources by forest managers may have concurred in reducing differences among populations in certain functional traits.

Structural leaf traits did not show a common pattern across populations (Table 3). Variations in tissue density, mesophyll thickness, and stomatal features have been related to the influence exerted by environmental conditions on leaf economics via modulation of hydraulic conductance and carbon budget (Brodribb et al., 2013; Sack et al., 2013; Blonder et al., 2015). Thicker leaves (and mesophyll) have long been recognized as structural adaptive trait in dry environments (Vogel, 2009), generally resulting in reductions in light interception and carbon gain expressed on a tissue volume basis. However, thicker palisade layer is a common trait for efficient light interception in mesomorphic sun leaves (Marchi et al., 2008). As efficient light harvesting is expensive in terms of biomass investment in support tissues, the efficiency with which foliage can be supported is a major factor determining species ability to grow under constrained environments (e.g., Niinemets et al., 1998). Intrinsic limitations associated with too high costs for foliar support may constrain more those populations in environments with high irradiance (southern Europe), where carbon for foliage construction is relatively cheaper.

As a major effect of experimental conditions on plant traits was expected to determine the phenotypic plasticity of corresponding traits, a significant interaction between beech populations and greenhouse conditions would indicate that responses to drought depended on the origin of seed sources. Hence, it is important to notice that we considered plasticity of a given trait at the population level, as an average value of the trait across individuals from each population, rather than at the genotype level.

### Drought stress impacts

This experiment and the companion study (Bolte et al., 2016) demonstrated the high correlation between physiological responses and water availability in these beech populations. This is consistent with findings in other common garden experiments conducted on beech, which showed considerable differences among populations in adaptation to environmental conditions (Kreyling et al., 2012, 2014; Thiel et al., 2014). The effect of drought treatment did not elicit marked differences among populations as expected (see Peuke et al., 2002).

When beech seedlings were exposed to water constraints, they adopted stress-compensating mechanisms (e.g., reduction of leaf water potential, regulation of gas exchange, control of plant growth), which differed among populations (Knutzen et al., 2015). Water constraints significantly decreased photosynthetic rate and stomatal conductance to prevent plant dehydration plant (Table 2) (Peuke et al., 2002; Aranda et al., 2015). However, according to Tognetti et al. (1995), there was no clear relationship between plant response and population range, suggesting a variable sensitivity to drought. The limitation of gas diffusion through stomata, before any changes occur in plant water status, could suggest a control of leaf water loss toward isohydric behavior (Attia et al., 2015), thus preserving leaf hydration. It is also possible that plants with highly sensitive stomata (isohydric), closing at relatively high water potentials, but with even more drought-sensitive hydraulic system could show anisohydric behavior. In the present study, C_i_/C_a_-values increased with the reduction of water availability, indicating regulation capacity of water balance achieved by a slow stomatal reaction in response to a decrease in leaf water potential (Lawlor and Tezara, 2009). Yet, a reduction in leaf intrinsic WUE (A/g_s_) was observed. Drought sensitivity increased in the sequence France Montagne Noir (PV4) < Denmark (PV1) < Romania (PV5) < France Crecy (PV3), with 21, 30, 79, and 81.5% of reduction, respectively, in comparison with control plants. Photosynthesis was substantially reduced and eventually stopped under severe drought, which may affect metabolic patterns and the allocation of carbon to structural traits to varying degrees, ultimately translating into plant death (McDowell et al., 2011). In this framework, the performance of beech seedlings was probably determined by plant size, defining the attitude of a plant to photosynthesize, store nutrients, and mobilize resources, and to growth (e.g., Villar-Salvador et al., 2012). Inhibition of metabolism, with maintenance of light respiration, probably increased C_i_, defining a greater sensitivity of photosynthetic rate than light respiration to water deficit (Cornic and Fresneau, 2002). Plants that display isohydric characteristics have tight and continuous water potential homeostasis through stomatal control. They constantly regulate their water loss within a certain range to avoid damaging water deficits due to hydraulic failure (Buckley, 2005), though this may inhibit CO_2_ diffusion into leaves and increase the risk of carbon starvation. The identification of population-specific performance under decreasing SWA, in this as well as in other recent experiments (e.g., Aranda et al., 2015; Knutzen et al., 2015; Dounavi et al., 2016), helps defining the risk of or resilience to local mortality across the native range of beech. However, drought led to heterogeneous and variable response patterns in these populations.

Beech populations variably combined functional traits in response to drought, although there was not a well-defined gradient in drought tolerance among populations. The phenotypic behavior showed a straight reduction of functional processes in response to decreasing SWA. The decrease of leaf water potential with the consequent reduction of assimilation rate under water stress showed a conservative control of water loss in these beech populations (Figure 3), highlighting the capacity of the plant hydraulic system to regulate the supply of water to leaves (Attia et al., 2015). The shape parameter of this relationship described accurately the response dynamics of seedlings to changing SWA (Figure 4). Furthermore, the inflection point of the function allowed the detection of a SWA threshold, where the SWA-value that induced a sharp drop of gas exchange was 28.34% (calculated as the mean of SWA-values, Figure 4). The reduction of SWA induced also the reduction of PI and Chl index and the increase of F_0_; whereas, the F_v_/F_m_ was not substantially affected by water stress. Overall, these parameters showed relatively high values, illustrating the resilience of the photochemical apparatus with decreasing SWA in beech (but see García-Plazaola and Becerril, 2000). However, when seedlings reached leaf predawn water potential of −2 MPa, gas exchange approached zero, indicating high sensitivity of stomatal behavior in response to extreme drought (in agreement with Ψ_pd_-values as low as −1.76 MPa—Köcher et al., 2009). A parallel reduction in the activity of PSII reaction center, efficiency of light capture, and rate of electron transfer was observed in all populations, which may avoid permanent damage from photoinhibition if stress conditions were quickly released. PV2 maintained the highest values of ETR and NPQ, utilizing more absorbed light energy for photochemistry and allocating more light energy to NPQ pathways than the other populations. The reversible down-regulation of PSII photochemistry would contribute to an enhanced photo-protection in severely stressed beech seedlings (Gallé and Feller, 2007).

Genetic variability and phenotypic plasticity, at morphological, physiological, and phenological level, are the key factors for the adaptation to environmental constraints, and are supposed to be wide in species, such as beech, with ample distribution range (Bussotti et al., 2015). The issue of the genetic background on the enhanced tolerance to drought in beech has been discussed mostly because of the probable evolutionary adaptation of local population by selection processes, which allow only drought-tolerant individuals to inherit genetic and phenotypic traits to next generations (Hampe and Petit, 2005). This is reputed to exhibit the highest resistance to the environmental stress (Thiel et al., 2014; Aranda et al., 2015; Knutzen et al., 2015). It must be pointed out that differences in moisture availability at the native sites may not be high enough to elicit distinct seedling performance among populations, and that factors other than differences in precipitation amount at the origin may be equally important, thus hindering the detection of clear population differences in common garden experiments.

## Concluding remarks

A common trend in responses of beech originated from different sites was established in relation to a decreasing of soil water availability. Phenotypic changes of plant traits in relation to SWA thresholds highlighted that decreasing SWA thresholds may favor ecophysiological traits that optimize carbon assimilation to counterbalance the reduced growing period. Functional traits define the contribution of genetic variability and phenotypic plasticity for understanding the adaptive potential to drought stress of beech across its large geographical range of distribution. In this sense, our study did not include populations spanning the entire environmental gradient of beech, which warrants further studies to better elaborate the relationships between ecophysiological acclimation to drought and seed source climate. A deeper understanding of the mechanism of drought tolerance in beech is needed to support strategies of forest management toward assisted selection and seed transfer. Planting of more resistant genotypes into drought-impacted forest stands may help implementing adaptive forest management options to ameliorate the impact to anticipated climate change, but parallel garden experiments are needed and should include more seed sources and ecophysiological traits.

## Author contributions

Substantial contributions to the conception and design of the work: CC, TC, AB, RT. Acquisition, analysis, or interpretation of data for the work: CC, MM, EP, LD, SM, LM, AA, TC, AB, RT. Drafting the work or revising it critically for important intellectual content: CC, MM, EP, LD, SM, LM, AA, TC, AB, RT. Final approval of the version to be published: CC, MM, EP, LD, SM, LM, AA, TC, AB, RT. Agreement to be accountable for all aspects of the work in ensuring that questions related to the accuracy or integrity of any part of the work are appropriately investigated and resolved: CC, MM, EP, LD, SM, LM, AA, TC, AB, RT.

### Conflict of interest statement

The authors declare that the research was conducted in the absence of any commercial or financial relationships that could be construed as a potential conflict of interest.
